# Supplementary material for: Polymorphism and Conformational Equilibrium of Nitro-Acetophenone in Solid State and under Matrix Conditions
Source: Molecules. 2021 May 22;26(11):3109. doi: 10.3390/molecules26113109 (PMC8197010; doi:10.3390/molecules26113109)
Supplement: Supplementary file 1 [file molecules-26-03109-s001.zip › molecules-1236445-supplementary.pdf]

Supplementary Material

Polymorphism and Conformational Equilibrium of Nitro-Acetophenone in Solid State and under Matrix Conditions

Łukasz Hetmańczyk [1], Przemysław Szklarz [2], Agnieszka Kwocz [2], Maria Wierzejewska [2], Magdalena Pagacz-Kostrzewa [2], Mikhail Ya. Melnikov [3], Peter M. Tolstoy [4] and Aleksander Filarowski [2,5,*]

1 Faculty of Chemistry, Jagiellonian University, Gronostajowa 2, 30-387 Kraków, Poland; hetmancz@chemia.uj.edu.pl
2 Faculty of Chemistry, Wrocław University, I14 F. Joliot-Curie st., 50-383 Wrocław, Poland; przemyslaw.szklarz@chem.uni.wroc.pl (P.S.); agnieszka.kwocz@chem.uni.wroc.pl (A.K.); maria.wierzejewska@chem.uni.wroc.pl (M.W.); magdalena.pagacz-kostrzewa@chem.uni.wroc.pl (M.P.-K.)
3 Department of Chemistry, Moscow State University, F. Joliot-Curie 14, 119991 Moscow, Russia; melnikov46@mail.ru
4 Institute of Chemistry, St. Petersburg State University, Universitetskij pr. 26, 198504 St. Petersburg, Russia; peter.tolstoy@spbu.ru
5 Frank Laboratory of Neutron Physics, Joint Institute of Nuclear Research, 141980 Dubna, Russia
* Correspondence: aleksander.filarowski@chem.uni.wroc.pl; Tel.: +48-71-3757283

Figure S1. Schemes of possible pathways of the N=O···H–O (**A**) O–H···O=C (**B**) equilibrium. Potential energy profile for internal rotation was calculated performing a relaxed scan on the DFT (B3LYP)/6-311++G(3df,3pd) PES along the relevant reaction coordinate.

Approach 1.

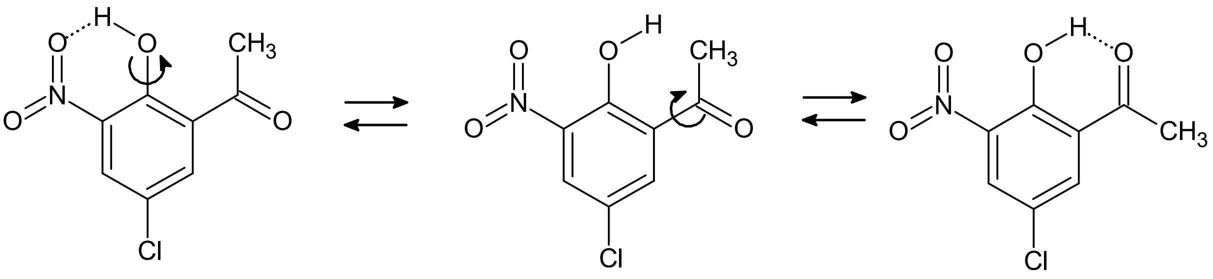

A **AB(TS1)** **B**

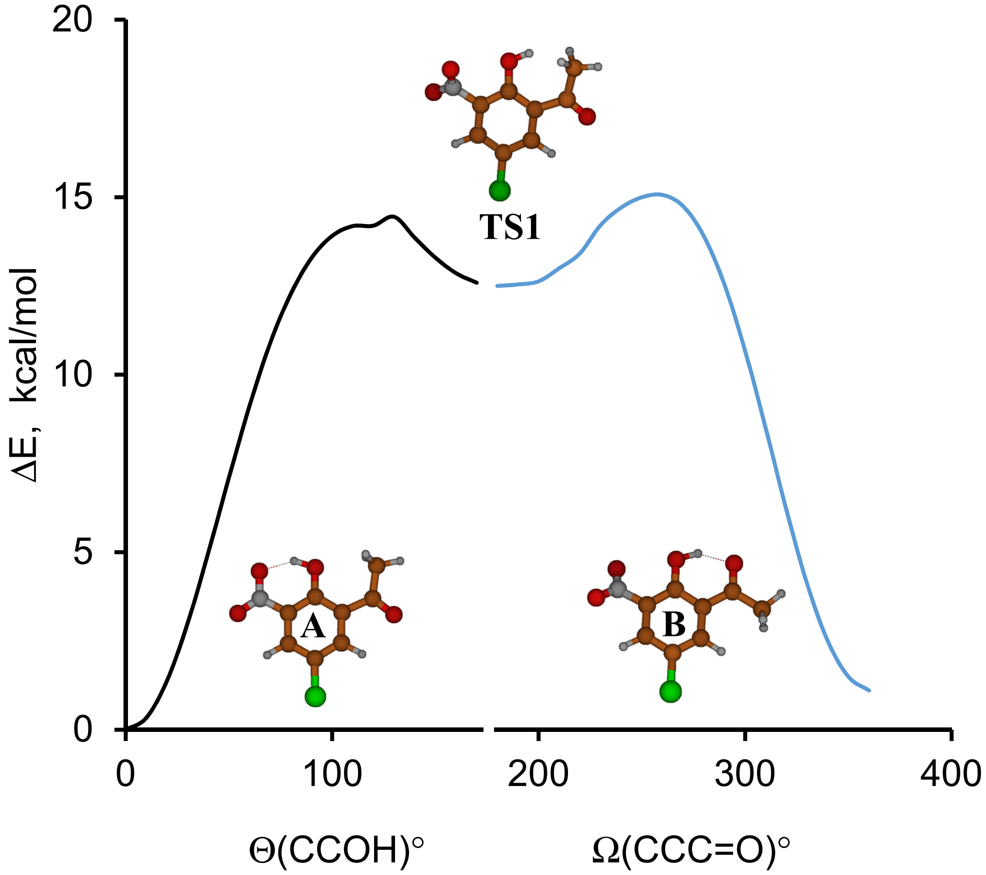

Figure S1 (continuation).

Approach 2.

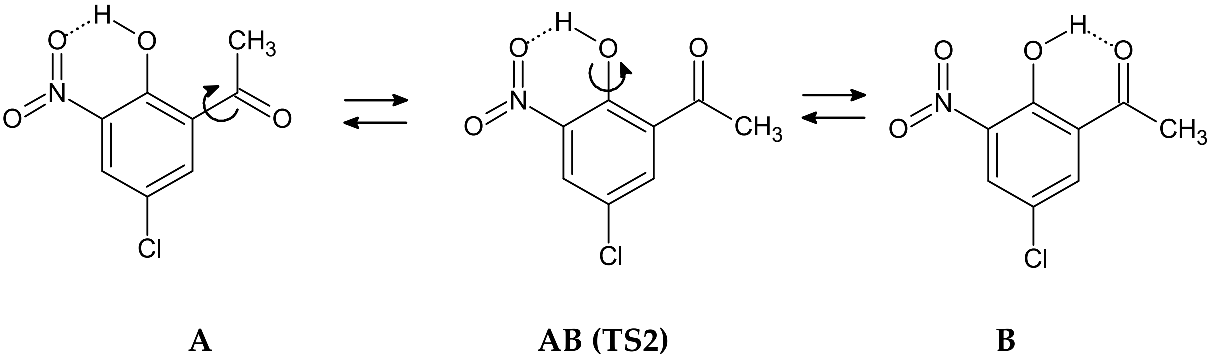

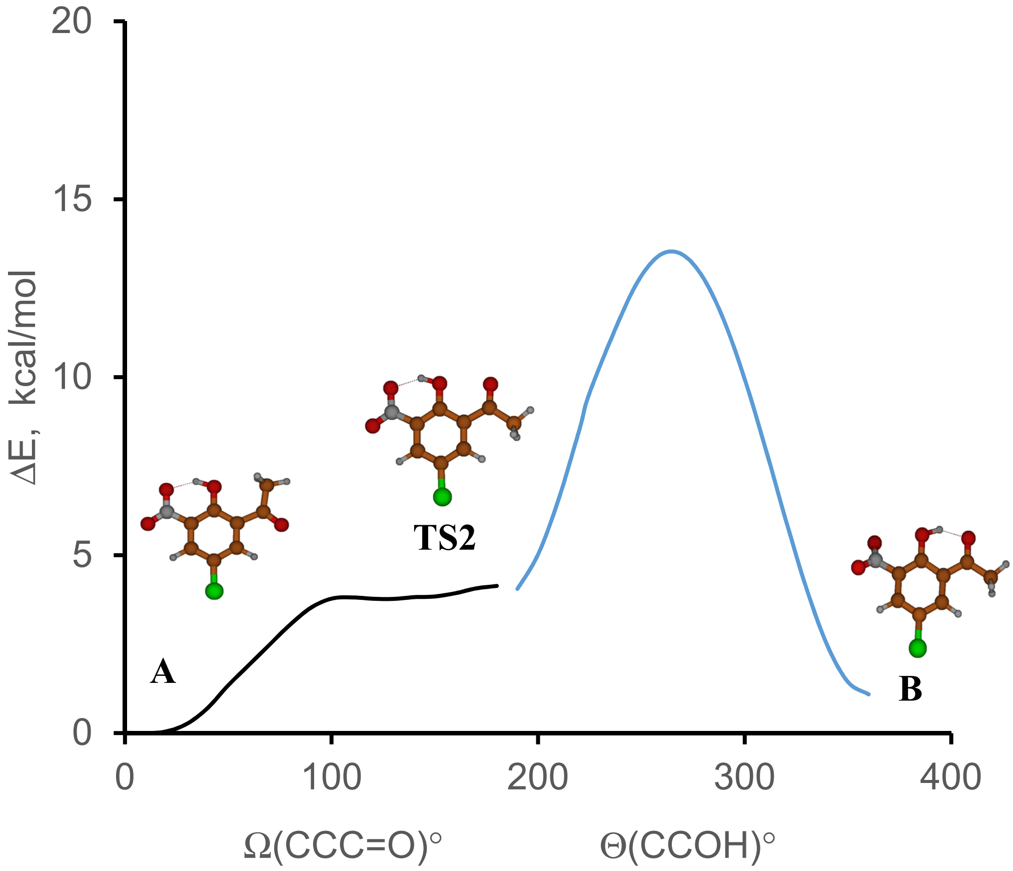

Figure S1 (continuation).

Approach 3.

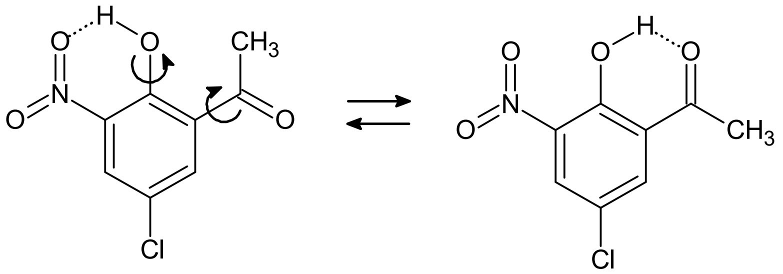

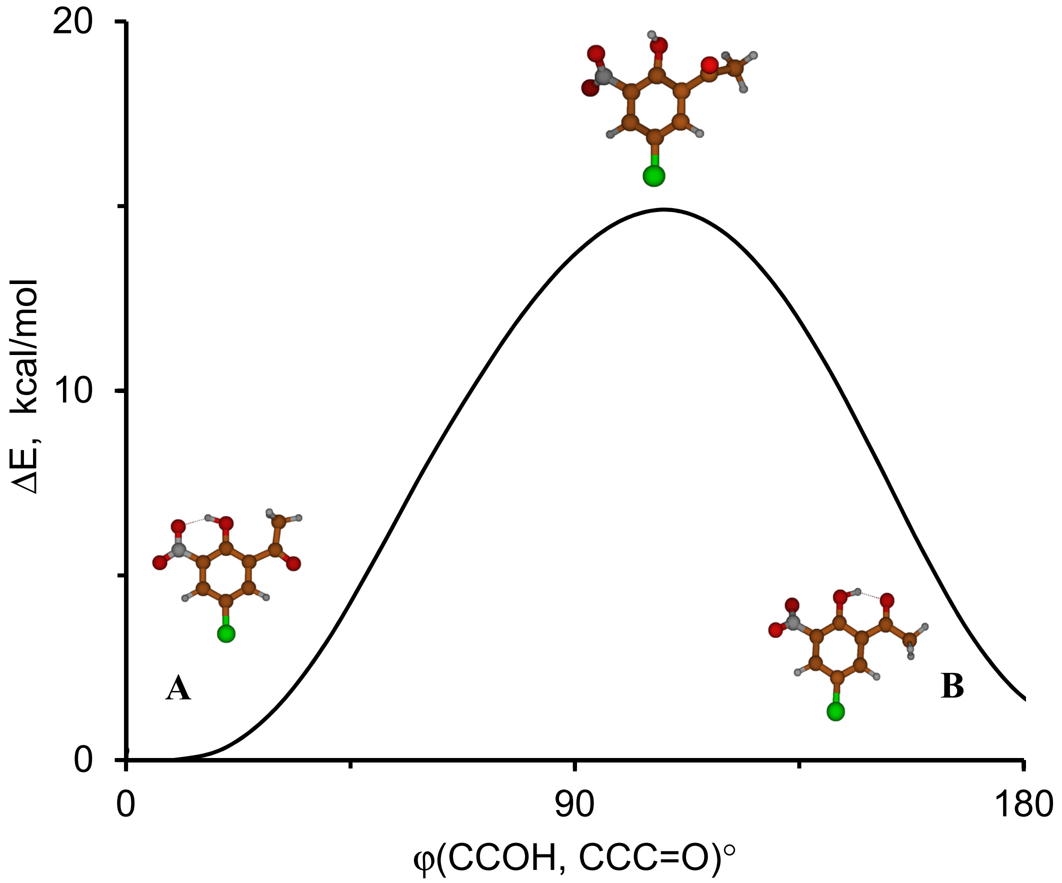

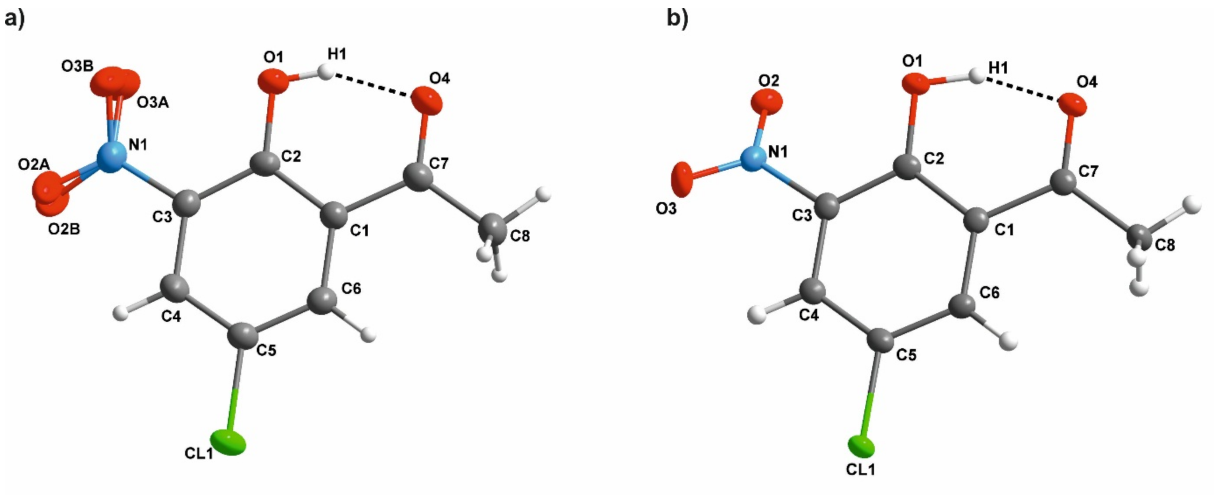

Figure S2. The X-ray structure and atom labelling schemes of two polymorphs of 5-chloro-3-nitro-2-hydroxyacetophenone (**a**—polymorph **I** and **b**—polymorph **II**). The intramolecular hydrogen bonding is shown as a broken line.

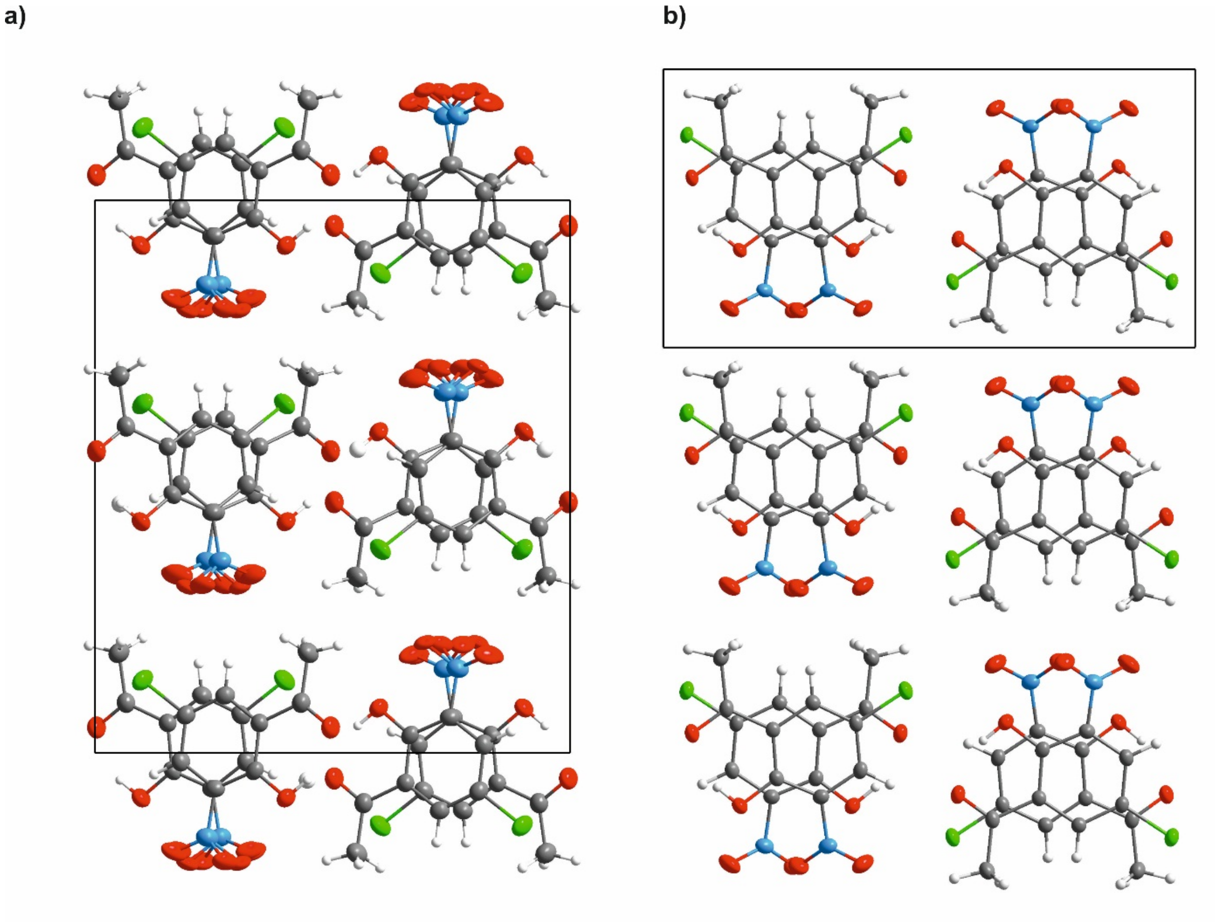

Figure S3. Crystal cells of **CNK** packing of polymorph **I** (**a**) and polymorph **II** (**b**).

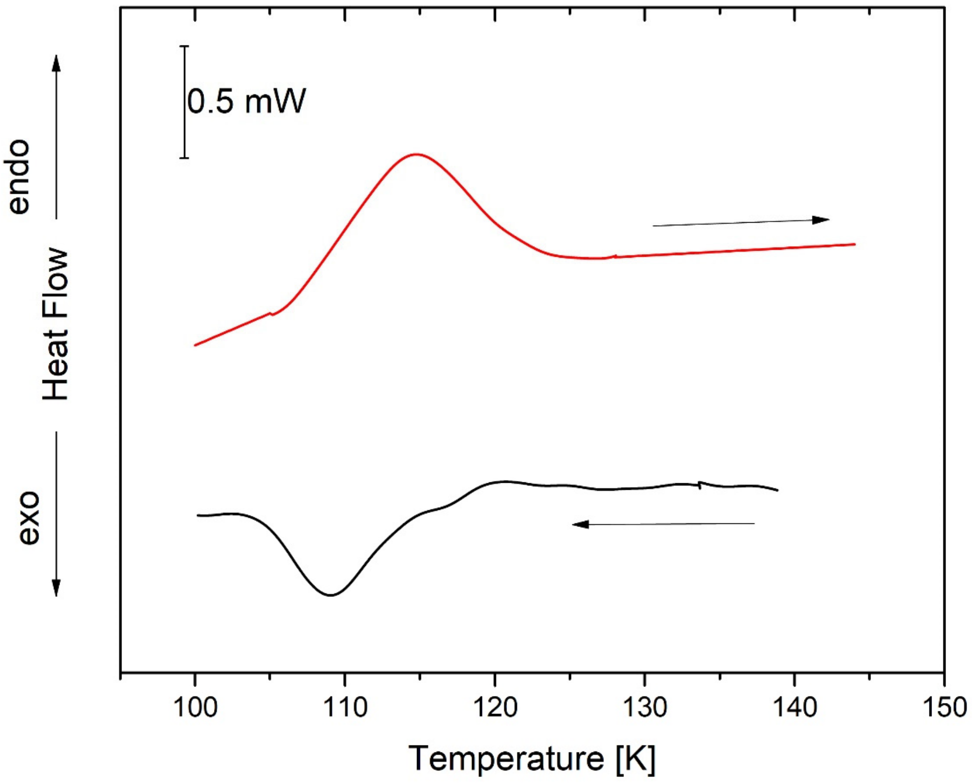

Figure S4. DSC cooling and heating runs for polymorph **I** of **CNK** compound (a ramp rate of 20 K/min). The traces show only one phase transition near 109.9 K (cooling) and 114.5 K (heating).

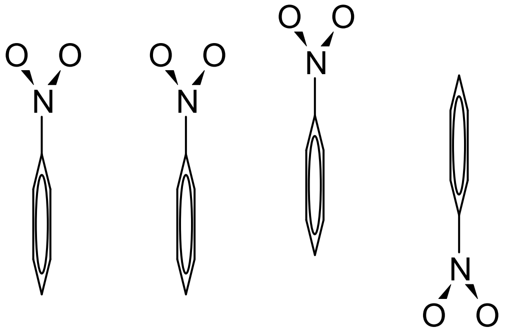

CNK **MNK**

Figure S5. The scheme of nitro group positions for 5-chloro-3-nitro-2-hydroxyacetophenone (**CNK**) and 5-methyl-3-nitro-2-hydroxyacetophenone[22] (**MNK**) in crystal cell.

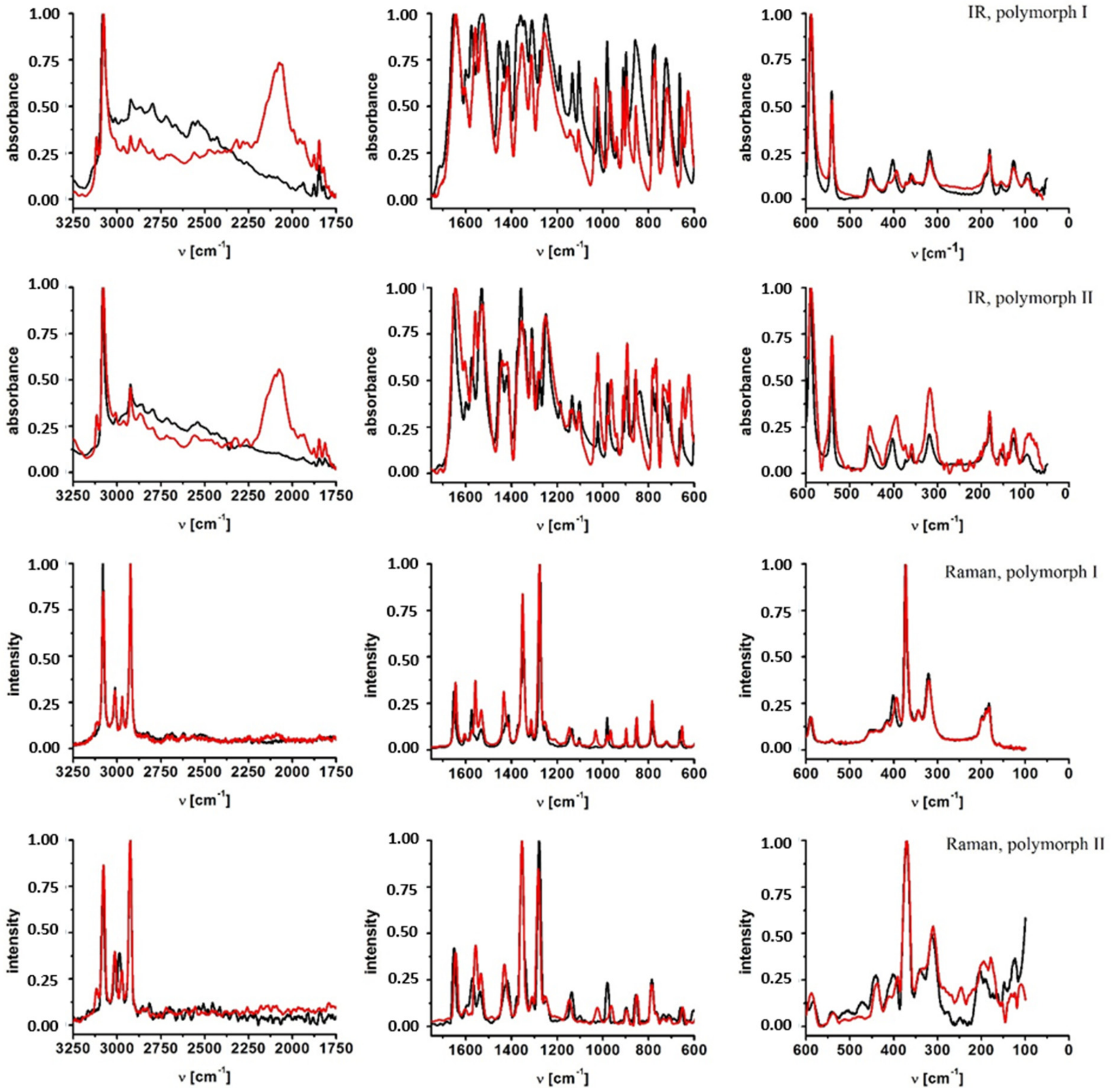

Figure S6. IR and Raman spectra (in the solid state) of both polymorphs of 5-chloro-3-nitro-2-hyroxyacetophenone (black line) and its deuterated analogue (red line).

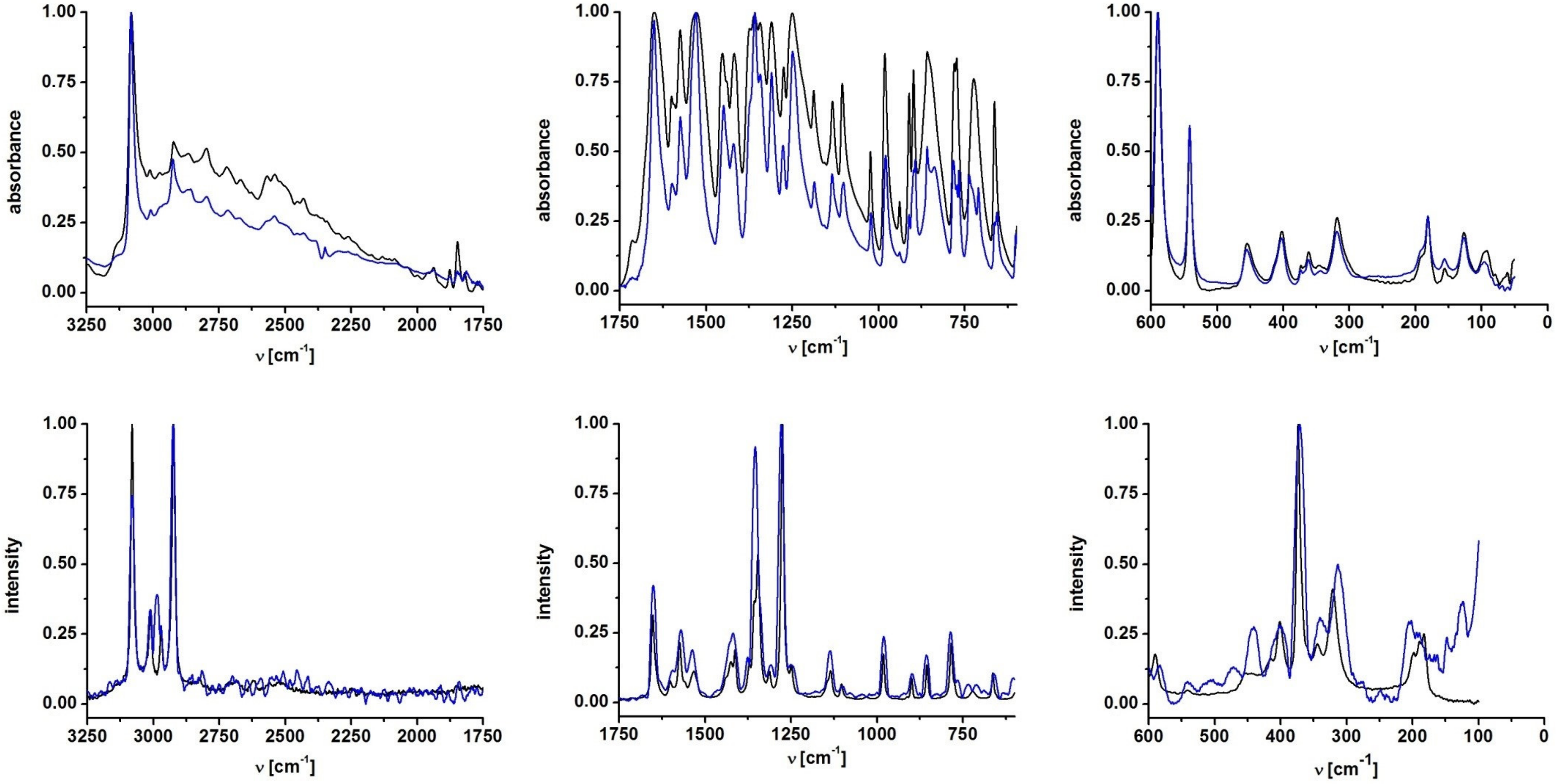

Figure S7. IR (**upper**) and Raman (**bottom**) spectra of polymorphs **I** (blue lines) and **II** (black lines).

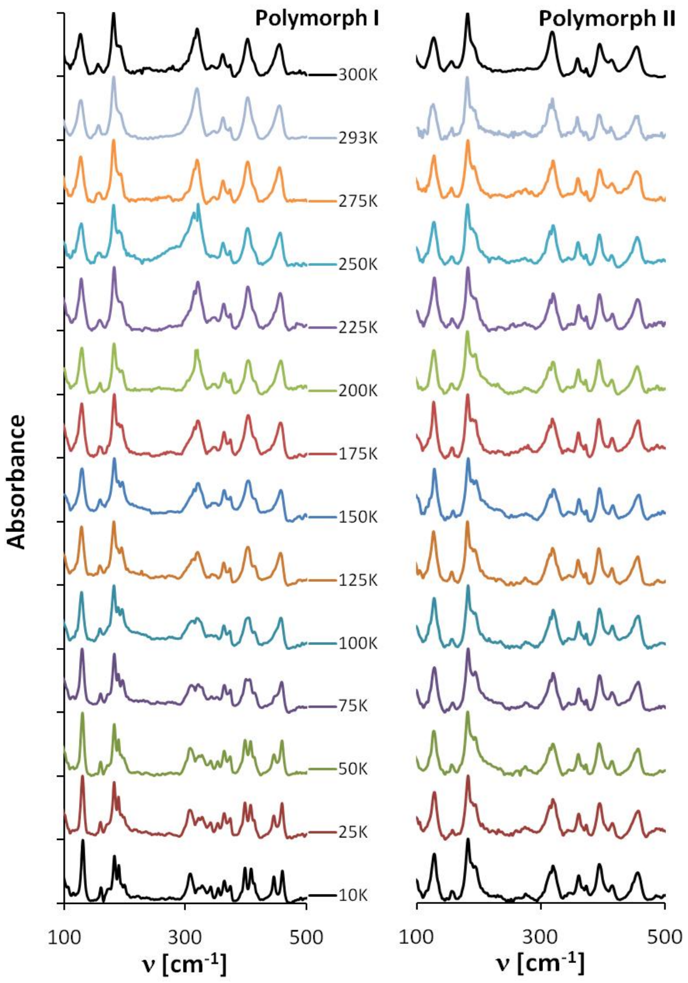

Figure S8. Infrared spectra of the **CNK** polymorphs recorded in the solid state from T = 300 to T = 10 K.

Table S1. Definitions of the internal coordinates used in the potential energy distribution (PED) analysis for the assignments of the vibrational spectra.

ν(CC)—ring stretching
ν(C_{ar}H)—stretching of the C_{aryl}–H bond
ν(C_{alk}H)—stretching of the C_{alkyl}–H bond
ν(C=O)—stretching of the C_{alkyl}=O bond
ν(OH)—stretching of the O–H bond
ν(OD)—stretching of the O–D bond
$\nu^{a}(NO_2)$ asymmetric stretching of the nitro group
$\nu^{s}(NO_2)$ symmetric stretching of the nitro group
ν(CO)—stretching of the C_{aryl}–O bond
ν($C_{ar}C_{alk}$)—stretching of the C_{aryl}–C_{alkyl} bond
ν($C_{alk}C_{alk}$)—stretching of the C_{alkyl}–C_{alkyl} bond
ν(C–N)—stretching of the C_{aryl}–N bond
ν(CCl)—stretching of the C_{aryl}–Cl bond
α(CC)—in-plane bending of the phenyl ring
δ(CCC_{alk})—in-plane bending of the C_{aryl}=C_{aryl}–C_{alkyl} chain
δ(CH)—in-plane bending of the C_{aryl}=C_{aryl}–H chain
δ(COH)—in-plane bending of the C_{aryl}–O–H chain
δ(COD)—in-plane bending of the C_{aryl}–O–D chain
δ(CCO)—in-plane bending of the C_{aryl}=C_{aryl}–O chain
δ(CCl)—in-plane bending of the C_{aryl}=C_{aryl}–Cl chain
δ(CCH_3)—in-plane bending of the C_{alkyl}–CH_3 unit
δ(NO_2)—in-plane bending of the C–NO_2 chain
δ(CC=O)—in-plane bending of the C_{aryl}–C_{alkyl}=O chain
δ(CCN)—in-plane bending of the C_{aryl}=C_{aryl}–N unit
δ($CC_{alk}C_{alk}$)—in-plane bending of the C_{aryl}–C_{alkyl}–C_{alkyl} chain
γ(COH)—out-of-plane bending of the O–H bond (the change in angle between the O–H and C–O bonds)
γ(COD)—out-of-plane bending of the O–D bond (the change in angle between the O–H and C–O bonds)
γ(CCN)—out-of-plane bending of the C–N bond (the change in angle between the C_{aryl}–N and C_{aryl}=C_{aryl} bonds)
γ(CNO_2)—out-of-plane bending of the NO bond (the change in angle between the C_{aryl}–N and N=O bonds)
γ(CCC_{alk})—out-of-plane bending of the C_{aryl}–C_{alkyl} (the change in angle between the C_{aryl}–C_{alkyl} bond and plane defined by three carbon atoms in the phenyl ring)
γ(CCH)—out-of-plane bending of the C_{aryl}–H (the change in angle between the C_{aryl}–H bond and plane defined by three carbon atoms in the phenyl ring)
γ(CCO)—out-of-plane bending of the C_{aryl}–O (the change in angle between the C_{aryl}–O bond and plane defined by three carbon atoms in the phenyl ring)
γ(CC=O)—out-of-plane bending of the C_{alkyl}=O (the change in angle between the C_{alkyl}=O bond and plane defined by three carbon atoms)
γ(CCCl)—out-of-plane bending of the C_{aryl}–Cl (the change in angle between the C_{aryl}–Cl bond and plane defined by three carbon atoms in the phenyl ring)
τ(CC)—torsion angle in the ring (change in the dihedral angle between two C_{aryl}=C_{aryl}=C_{aryl} planes in the phenyl ring)
τ(CH_3)—torsion around the C_{alkyl}–C_{alkyl} bond
τ(NO_2)—torsion around the C_{alkyl}–N bond
τ(CC_{alk})—torsion angle in the ring (change in the dihedral angle between C_{aryl}=C_{aryl}=C_{aryl} and C_{aryl}=C_{aryl}–C_{alkyl} planes)

Table S2. Experimental infrared (IR_{exp}), Raman (R_{exp}) and calculated DFT (6-311++G(2d,2p)) spectroscopic data for 5-chloro-3-nitro-2-hydroxyacetophenone (polymorphs **I** and **II**) and its mono deuterated derivative (OD) in the solid state at different temperatures. Potential Energy Distribution (PED) calculated with GAR2PED program.[42]

**IR_{exp}**	**-**	**-**	**-**	**-**	**-**	**R_{exp}**	**-**	**R_{exp}**	**-**	**DFT**	**DFT**	**DFT**	**PED**
**I**	**-**	**II**	**-**	**I**	**-**	**I**	**-**	**II**	**-**				
**300 K**	**300 K**	**300 K**	**300 K**	**5 K**	**5 K**	**300 K**	**300 K**	**300 K**	**300 K**	**Freq.**	**IR(A)**	**R(I)**	
**OH**	**OD**	**OH**	**OD**	**OH**	**OD**	**OH**	**OD**	**OH**	**OD**	**OH**			
3138		3138w											
	3117w		3116		3119w		3115w		3115m	3127			
3081s	3077s	3083s	3078	3083s	3079s	3080m	3078m	3080	3077w	3119	12.2	52.5	ν(C_{ar}H) 99
3011w	3011sh		3015	3012w		3011w	3013w	3011	3012w	3057	0.1	51.2	ν(C_{ar}H) 99
	2964w	2968w	2968			2971w	2971w	2975	2973w	3006	0.1	115.8	ν(C_{alk}H) 99
2921w	2923w	2924w	2924	2921w	2922w	2925s	2922s	2925	2925s	3006	3.3	46.3	ν(C_{alk}H) 99
	2866w		2866		2871w					2944	0.8	161.0	ν(C_{alk}H) 100
3000-2100vb		3000-2100vb		3000-2100vb						3077	491.4	113.3	ν(OH) 98
	2076vb		2075vb		2078vb								ν(OD)*
1650s	1644s	1650s	1644s	1651s	1645s	1652s	1644s	1650s	1643s	1664	291.6	62.6	ν(C=O) 52, δ(CCC_{alk}) 8
1601w	1603s	1600w	1604s	1601w	1604w	1600w	1605w	1596m	1603w	1595	50.0	9.8	ν(CC) 45, ν(C=O) 13, $ν^a$(NO_2) 7, δ(CH) 7
1575s	1575w	1575s	1577sh	1577s	1576w	1574m	1575m	1570m		1563	110.5	48.6	ν(CC) 45, δ(COH) 19, δ(CCO) 7, δ(CH) 6, α(CC) 6
	1558s		1558s	1559w	1559s	1560w	1558s		1556s				
1530s	1526s	1528s	1528s	1528s	1527s	1533w	1531m	1540m	1532s	1551	213.3	22.7	$ν^{as}$(NO_2) 68, ν(CC) 6
1452s		1450s		1453s	1452sh					1433	205.0	9.0	ν(CC) 27, δ(COH) 19, ν(CO) 16, δ(CH) 10
	1438s		1439s	1439sh	1440s	1443sh							δ(COD)*
						1425m	1433s	1419m	1430s	1432	13.6	9.8	δ(CCH_3) 94
1418s	1417s	1418s	1418s	1418s	1418s	1413m	1413sh	1412m	1410w	1422	58.1	15.4	δ(CCH_3) 70, ν(CO) 6
										1407	3.1	33.5	ν(CC) 30, ν(C=O) 11, ν(CO) 9, δ(COH) 7, δ(CCH_3) 7, δ(CCC_{alk}) 5
1374w	1375sh	1374sh		1375m	1374sh	1372w		1378m	1377sh	1372	101.5	21.3	ν(CC) 24, δ(COH) 22, δ(CH) 14, ν($C_{ar}C_{alk}$) 5
										1353	54.4	5.6	δ(CCH_3) 66, ν($C_{alk}C_{alk}$) 11
1360s	1354s	1360s	1355s	1361s	1355s	1357s	1351s	1354s	1353s	1333	223.8	99.8	$ν^s$(NO_2) 49, ν(C-N) 17, δ(NO_2) 13, δ(CH) 5
1341w		1341w		1344w		1346s		1339sh					

1310s	1312s	1310s	1311s	1311s	1314s	1313w	1313w	1308w	1310w	1302	61.6	13.6	ν(CC) 40, ν(C$_{ar}$C$_{alk}$) 12, δ(CC=O) 11, δ(CCH$_3$) 9, δ(CCC$_{alk}$) 6
								1299w					
1275s	1282sh	1275s	1281s	1276w	1282sh	1274s	1277s	1281s	1281s	1284	37.9	83.9	ν(CO) 29, ν(CC) 34, δ(CH) 12
1251s	1257s	1250s	1250s	1252s	1258s	1249w	1253w	1248w	1248w	1238	258.5	14.4	ν(C$_{ar}$C$_{alk}$) 22, δ(CH) 17, ν(CC) 29, ν(C$_{alk}$C$_{alk}$) 6
1187m	1190sh	1187m	1185sh	1189m	1190w					1170	1.1	5.6	δ(CH) 32, ν(CC) 24, δ(CCO) 10, ν(C-N) 8, δ(COH) 6
	1143w		1143w		1145w		1146w		1145w				
1133s	1133w	1134m	1134w	1134m	1134w	1134w	1135w	1137m		1112	29.6	31.8	ν(CC) 32, δ(CH) 18, ν(CCl) 12, ν(C$_{alk}$C$_{alk}$) 6
1106s	1107m	1101m	1105w	1106m	1107w	1103w	1104w	1096w		1078	35.0	4.9	α(CC)21, ν(CC)17, δ(CCH$_3$)10, ν(C-N)9, δ(CH)8, ν(C$_{alk}$C$_{alk}$)7, δ(CH)6
-	1032s	-	1032sh	1035w	1034s	1031w	1031w						
1023s	1023s	1021m	1022s	1024m	1024m				1025w	1010	1.9	0.1	δ(CCH$_3$) 68, δ(CC$_{alk}$C$_{alk}$) 18
981s	980sh	980m	980w	982s	982w	981s	981w	980s		952	57.5	13.8	δ(CCH$_3$) 45, ν(C$_{alk}$C$_{alk}$) 17, ν(C$_{ar}$C$_{alk}$) 5, δ(CC=O) 5
	968s		963s	967sh	968s		965w		963w				-
940w	939m	940w	940w	940w	940w					906	2.9	0.1	γ(CCH) 83, τ(CC) 7
911s	911s	912w	911m	912s	912s		912w						
899s	896s	894s	894s	899s	897s		898w		895w	880	10.3	6.7	δ(NO$_2$) 19, ν(C-N) 18, α(CC) 15, ν(CCl) 10
										872	47.1	0.4	γ(CCH) 69, τ(CC) 15
860b	856s	860s	856s	860b	857s	856m	852m	855m	851m	869	66.9	0.7	γ(COH) 86,* γ(CCH) 7
860b		837s	835w	860b						840	16.9	8.4	α(CC) 33, ν(C$_{alk}$C$_{alk}$) 15, ν(CCl) 10, ν(CC) 10, ν(C$_{ar}$C$_{alk}$) 8, δ(NO$_2$) 5
781s	781s	782s	782s	781s	781s	785s	785s	786s	787s	771	20.2	13.5	γ(CNO$_2$) 18, δ(NO$_2$) 10, ν(C$_{ar}$C$_{alk}$) 7, ν(CC) 6, ν(CCl) 6, δ(CCN) 6, ν(CO) 5, γ(CCN) 5
773s	773	767s	767s	773m	773s			768w	768w	750	12.6	5.2	τ(CC) 30, γ(CCO) 24, γ(CNO$_2$) 12, γ(CCN) 12, γ(CCC$_{alk}$) 6
724s	719s	737s	737s	725m	721s	723w	723w	731w	733w	716	42.0	1.9	τ(CC) 23, γ(CCO) 17, δ(NO$_2$) 12, γ(CCC$_{alk}$) 9, γ(CNO$_2$) 7, α(CC) 6
		710m	709s					711w	711w	691	26.5	3.3	τ(CC) 20, γ(CCO) 14, γ(CCC$_{alk}$) 12, γ(CNO$_2$) 11, ν(CCl) 8, δ(NO$_2$) 7
663s	653s	657m	648s	664m	654m	664w	653w	658w	652w	644	24.3	4.2	δ(CCO) 16, δ(CC=O) 15, δ(C$_{ar}$C$_{alk}$) 10, δ(NO$_2$) 8, ν(C$_{alk}$C$_{alk}$) 7, γ(CCC$_{alk}$) 6
	625s		624s	633w	630m								γ(COD)*
		607w						606w		588	13.5	3.4	δ(CC$_{alk}$C$_{alk}$) 20, τ(CC) 18, δ(CC=O) 15, δ(CCH$_3$) 11, γ(CCC$_{alk}$) 8, γ(CCN) 6
588s	587s	583m	587s	589s	589s	590w	588w	583w	588w	568	12.9	1.9	τ(CC) 31, γ(CCCl) 14, δ(CC=O) 15, δ(CC$_{alk}$C$_{alk}$) 9, δ(CH$_3$) 6
540s	540s	540m	539s	540m	541m		541w			525	9.2	0.8	τ(CC) 46, γ(CCCl) 24, γ(CCO) 14
454w	453w			460w	457w	453w	453w			464	1.7	0.9	τ(CC) 22, δ(NO$_2$) 12, γ(CCN) 11, δ(CCO) 8, δ(CC=O) 7, γ(CCC$_{alk}$) 6, δ(CCC$_{alk}$)6
		441w	439w	445w				442w	438w				-

		415sh	414w	416w	416w			433	1.3	2.6	δ(CC=O) 18, τ(CC) 13, α(CC) 19, δ(CCO) 9, δ(CCC$_{alk}$) 7, ν(C$_{ar}$C$_{alk}$) 6
		409m									ν_{σ}(OHO)*
392w	387w	398w	393w		393w	395w	387s	398	0.3	0.6	τ(CC) 35, γ(CCC$_{alk}$) 17, δ(NO_2) 9
		374w	371w	375s	372s	371s	370s	380	4.0	2.8	α(CC) 18, δ(CC=O) 17, δ(CCO) 16, δ(NO_2) 7, τ(CC) 7, δ(CCl) 6
		364w	360w					357	0.9	10.6	ν(CCl) 38, α(CC) 21, ν(CC) 6, δ(CC=O) 5
		354w									
		341w		343w	343w	340w	335w	344	3.9	0.6	δ_{alk}(CCC$_{alk}$) 14, α(CC) 13, ν(C$_{ar}$C$_{alk}$) 11, δ(CCO) 9, δ(CCl) 8
		328w	321w	322m	320s			318	0.6	1.2	γ(CCCl) 20, γ(CCN) 17, τ(CC) 24, γ(CCC$_{alk}$) 10, γ(CCO) 8, δ(NO_2) 7
		308w				313w	310w	293	4.2	6.1	δ(CC=O) 28, ν(C-N) 14, δ(CCl) 12, α(CC) 6
						245	245w				
		200w	193w	199w	199w	203w					
		190w		190w	190w	194w	194w				
		182w	182w	182w	182w			175	0.8	2.4	τ(CC) 28, γ(CCC$_{alk}$) 17, γ(COH) 14, γ(CCN) 12, δ(CC$_{alk}$C$_{alk}$) 6, γ(CCH) 5
						175w	178w	169	1.6	0.7	δ(CCC$_{alk}$) 39, δ(CCN) 27, δ(CC=O) 5
		161w						164	1.7	2.2	δ(CCl) 49, δ(CCN) 25, δ(CCC$_{alk}$) 6
			155w					145	0.1	0.6	τ(CH_3) 75, δ(CCH_3) 11
		130w	127w					134	0.3	0.3	τ(CC) 32, τ(CC$_{alk}$) 16, γ(CCC$_{alk}$) 13, γ(COH) 13, γ(CCCl) 9
		106w						104	3.4	0.2	γ(COH) 31, τ(CC$_{alk}$) 28, γ(CCN) 14, γ(CCC$_{alk}$) 12, γ(CCCl) 6
		98w	98w								
		78w									
		74w	74w								
		62w						59	0.6	0.5	τ(CC$_{alk}$) 49, τ(CC) 34
		51w						42	0.7	2.4	τ(NO_2) 68, δ(CCO) 8, δ(CCN) 6

Abbreviations s, m, w, vb, b and sh mean strong, middle, weak, very broad, broad and shoulder of experimental bands, respectively.
*—data obtained on basis of frequency isotopic ratio analysis.

Table S3. Experimental infrared spectra measured under the matrix condition and calculated DFT (6-311++G(2d,2p)) spectroscopic data for 5-chloro-3-nitro-2-hydroxyacetophenone and its mono deuterated derivative (OD). Potential Energy Distribution (PED) calculated with GAR2PED program.[42]

IR_{exp}	IR_{exp}	DFT			PED	Conformer
10 K	10 K		IR(A)	R(A)		
OH	OD	OH				
3100w	3095w	3127	12.2	52.5	$\nu(C_{ar}H)$ 99	
3060w	3084w	3119	0.1	51.2	$\nu(C_{ar}H)$ 99	
3016w	3013w	3057	0.1	115.8	$\nu(C_{alk}H)$ 99	
2993w	2991w					
2941w	2940w	3006	3.3	46.3	$\nu(C_{alk}H)$ 99	
2890w	2900w	2944	0.8	161.0	$\nu(C_{alk}H)$ 100	
3000	2339	3077	491.4	113.3	ν(OH) /ν(OH) 100	
1700s	1700s	1646	291.6	62.6	ν(C=O) 52, $\delta(CCC_{alk})$ 8	**A**
1667s	1659s				-	**B**
1624w	1626w					
1615s	1612s	1595	50.0	9.8	ν(CC) 45, ν(C=O) 13, $\nu^a(NO_2)$ 7, δ(CH) 7	
1592s	1590w	1563	110.5	48.6	ν(CC) 45, δ(COH) 19, δ(CCO) 7, δ(CH) 6, α(CC) 6	
1571w	1572s					
1567w	1564s					
1550s	1548s	1551	213.3	22.7	$\nu^a(NO_2)$ 68, ν(CC) 6	**B**
1539s	1535s				-	**A**
1458s	1458s	1433	205.0	9.0	ν(CC) 27, δ(COH) 19, ν(CO) 16, δ(CH) 10	
1449s	1449s					
1436s	1433s	1432	13.6	9.8	$\delta(CCH_3)$ 94	
1418w	1419w	1422	58.1	15.4	$\delta(CCH_3)$ 70, ν(CO) 6	
	1401s	1407	3.1	33.5	ν(CC) 30, ν(C=O) 11, ν(CO) 9, δ(COH) 7, $\delta(CCH_3)$ 7, $\delta(CCC_{alk})$ 5	
1374m	1363s	1372	101.5	21.3	ν(CC) 24, δ(COH) 22, δ(CH) 14, $\nu(C_{ar}C_{alk})$ 5	
		1353	54.4	5.6	$\delta(CCH_3)$ 66, $\nu(C_{alk}C_{alk})$ 11	
1356s	1358s	1333	223.8	99.8	$\nu^s(NO_2)$ 49, ν(C-N) 17, $\delta(NO_2)$ 13, δ(CH) 5	**B**
1346s	1335s					
1316m	1316m	1302	61.6	13.6	ν(CC) 40, $\nu(C_{ar}C_{alk})$ 12, δ(CC=O) 11, $\delta(CCH_3)$ 9, $\delta(CCC_{alk})$ 6	
1303s	1304s				$\nu^s(NO_2)$ 49, ν(C-N) 17, $\delta(NO_2)$ 13, δ(CH) 5	**A**
1288w	1267sh	1284	37.9	83.9	ν(CO) 29, ν(CC) 34, δ(CH) 12	
1269s	1254w				δ(COH)*	**B**
1243s	1246s	1238	258.5	14.4	$\nu(C_{ar}C_{alk})$ 22, δ(CH) 17, ν(CC) 29, $\nu(C_{alk}C_{alk})$ 6	
	1229w					
1211w	1212w					
	1201w					
1188w		1170	1.1	5.6	δ(CH) 32, ν(CC) 24, δ(CCO) 10, ν(C-N) 8, δ(COH) 6	
1166s	1166w				δ(COH)*	**A**
1136w						
1124s	1132m	1112	29.6	31.8	ν(CC) 32, δ(CH) 18, ν(CCl) 12, $\nu(C_{alk}C_{alk})$ 6	
1104w	1110w	1078	35.0	4.9	α(CC) 21, ν(CC) 17, $\delta(CCH_3)$ 10, ν(C-N) 9, δ(CH) 8, $\nu(C_{alk}C_{alk})$	

					7, δ(CH) 6	
1100w	1096w					
1086w						
1028w	1028m	1010	1.9	0.1	δ(CCH$_3$) 68, δ(CC$_{alk}$C$_{alk}$) 18	
976	976m	952	57.5	13.8	δ(CCH$_3$) 45, ν(C$_{alk}$C$_{alk}$) 17, ν(C$_{ar}$C$_{alk}$) 5, δ(CC=O) 5	
	959m				δ(COD)*	**B**
	942w					
907	906w	906	2.9	0.1	γ(CCH) 83, τ(CC) 7	
898w	895s	880	10.3	6.7	δ(NO$_2$) 19, ν(C-N) 18, α(CC) 15, ν(CCl) 10	
	895s				δ(COD)*	**A**
859w	857s	872	47.1	0.4	γ(CCH) 69, τ(CC) 15	
		840	16.9	8.4	ν(C$_{alk}$C$_{alk}$) 15, α(CC) 33, ν(CCl) 10, ν(CC) 10, ν(C$_{ar}$C$_{alk}$) 8, δ(NO$_2$) 5	
823w		869	66.9	0.7	γ(COH)* 86, γ(CCH) 7	**B**
788w	787m	771	20.2	13.5	γ(CNO$_2$) 18, δ(NO$_2$) 10, ν(C$_{ar}$C$_{alk}$) 7, ν(CC) 6, ν(CCl) 6, δ(CCN) 6, ν(CO) 5, γ(CCN) 5	
772s	772s	750	12.6	5.2	τ(CC) 30, γ(CCO) 24, γ(CCN) 12, γ(CNO$_2$) 12, γ(CCC$_{alk}$) 6	
736s	731s	716	42.0	1.9	τ(CC) 23, γ(CCO) 17, δ(NO$_2$) 12, γ(CCC$_{alk}$) 9, γ(CNO$_2$) 7, α(CC) 6	
	719w				γ(COH)*	**A**
711	708w	691	26.5	3.3	τ(CC) 20, γ(CCO) 14, γ(CCC$_{alk}$) 12, γ(CNO$_2$) 11, ν(CCl) 8, δ(NO$_2$) 7	
661w	653w	644	24.3	4.2	δ(CCO) 16, δ(CC=O) 15, δ(C$_{ar}$C$_{alk}$) 10, δ(NO$_2$) 8, ν(C$_{alk}$C$_{alk}$) 7, γ(CCC$_{alk}$) 6	
648w	636w					
	619w				γ(COD)*	**B**
604m	604m	588	13.5	3.4	δ(CC$_{alk}$C$_{alk}$) 20, τ(CC) 18, δ(CC=O) 15, δ(CCH$_3$) 11, γ(CCC$_{alk}$) 8, γ(CCN) 6	
591m	591s	568	12.9	1.9	τ(CC) 31, γ(CCCl) 14, δ(CC=O) 15, δ(CC$_{alk}$C$_{alk}$) 9, δ(CH$_3$) 6	
583w	587w					
	579m					
549w		525	9.2	0.8	τ(CC) 46, γ(CCCl) 24, γ(CCO) 14	
	534s				γ(COD)*	**A**

Abbreviations s, m and w mean strong, middle and weak bands, respectively. *—data obtained on basis of frequency isotopic ratio analysis.

Table S4. Experimental incoherent inelastic neutron scattering and calculated DFT data for **CNK** and mono deuterated (**CNK-OD**) derivative. Distribution (PED) calculated with GAR2PED program.[42]

CNK	CNK-OD	Δfreq.	DFT	PED
1202w	1186w		1238	ν(C$_{ar}$C$_{alk}$) 22, δ(CH) 17, ν(CC) 29, ν(C$_{alk}$C$_{alk}$) 6
1156m	1171m		1170	δ(CH) 32, ν(CC) 24, δ(CCO) 10, ν(C-N) 8, δ(COH) 6
	1127m	44		-
1099w	1085w		1112	α(CC) 21, ν(CC) 17, δ(CCH_3) 10, ν(C-N) 9, δ(CH) 8, ν(C$_{alk}$C$_{alk}$) 7, δ(CH) 6
1045m	1032m		1010	δ(CCH_3) 68, δ(CC$_{alk}$C$_{alk}$) 18
996m	996w		952	δ(CCH_3) 45, ν(C$_{alk}$C$_{alk}$) 17, ν(C$_{ar}$C$_{alk}$) 5, δ(CC=O) 5
949m	949m		906	γ(CCH) 83, τ(CC) 7
916m	927m	22		-
916m			869	γ(COH) 86,* γ(CCH) 7
895m		21		-
875sh	865w		872	γ(CCH) 69, τ(CC) 15
860sh	856w			
837w	827w			
791w	783w		771	γ(CNO_2) 18, δ(NO_2) 10, ν(C$_{ar}$C$_{alk}$) 7, ν(CC) 6, ν(CCl) 6, δ(CCN) 6, ν(CO) 5, γ(CCN) 5
766w			750	τ(CC) 30, γ(CCO) 24, γ(CNO_2) 12, γ(CCN) 12, γ(CCC$_{alk}$) 6
742w	742w			
	727w			γ(COD)*
712w	712w			
	704w			
675w			644	δ(CCO) 16, δ(CC=O) 15, δ(C$_{ar}$C$_{alk}$) 10, δ(NO_2) 8, ν(C$_{alk}$C$_{alk}$) 7, γ(CCC$_{alk}$) 6
	675vw			γ(COD)*
	656w	19		
600m	594m		588	δ(CC$_{alk}$C$_{alk}$)20, τ(CC)18, δ(CCH_3)11, δ(CC=O) 15, γ(CCC$_{alk}$)8, γ(CCN)6
545m	546m	55/48		-
519w	516w			
502w				
467m	463m		464	τ(CC)22, δ(NO_2)12, γ(CCN)11, δ(CCO)8, δ(CC=O)7, γ(CCC$_{alk}$)6, δ(CCC$_{alk}$) 6
448m	448m	19/15		-
411m	418m			ν$_{σ}$(OHO)*
405m	398m			-
375m	370m			-
367m	355m			-
336	336		357	ν(CCl) 38, α(CC) 21, ν(CC) 6, δ(CC=O) 5
309	309	27		-
279w	279w			
202w	200w		175	τ(CC) 28, γ(CCC$_{alk}$) 17, γ(COH) 14, γ(CCN) 12, δ(CC$_{alk}$C$_{alk}$) 6, γ(CCH) 5
176s	176s		145	τ(CH_3) 75, δ(CCH_3) 11
165	165			lattice modes
106	105			-
94	95			-
62	62			-
50	50			-

Abbreviations s, m and w mean strong, middle and weak bands, respectively. *—data obtained on basis of isotopic effect.

Table S5. Crystal data and structure refinement for polymorphs **I** and **II**.

Compound	**Polymorph I**	**Polymorph II**	**Polymorph II**
Empirical formula		$C_8H_6ClNO_2$	
Formula weight		183.59	
Temperature [K]	200(2)	200(2)	100(2)
Wavelength[Å]		0.71073	
Crystal system		Monoclinic	Monoclinic
Space group	Pccn	P21/c	P21/c
Unit cell dimensions			
a [Å]	7.65370(10)	8.0648(3)	8.0378(4)
b [Å]	14.0388(3)	15.4442(6)	15.3927(8)
c [Å]	16.3184(2)	6.8597(3)	6.7766(3)
	90	90.752(3)	90.667(4)
Volume [Å^3]	1753.39(5)	854.33(6)	838.37(7)
Z	8	4	4
Calculated density [Mg/m^3]	1.391	1.427	1.455
Absorption coefficient [mm^{-1}]	0.392	0.402	0.410
F(000)	752	376	376
Crystal size [mm]			
Theta range for data collection [°]	2.496 to 26.800	3.250 to 36.744	2.534 to 24.285
Index ranges	−9 <= h <= 8<br>−17 <= k <= 14<br>−20 <= l <= 19	−12 <= h <= 10<br>−22 <= k <= 21<br>−8 <= l <= 11	9 <= h <= 9<br>−17 <= k <= 17<br>−7 <= l <= 7
Reflections collected/unique	7126/1842 [R(int) = 0.0583]	9641/3235 [R(int) = 0.1045]	4462/1311 [R(int) = 0.0598]
Data/restraints/parameters	1842/0/151	3235/0/132	1311/0/132
Goodness-of-fit on F^2	1.231	1.062	1.036
Final R indices [I>2sigma(I)]	R1 = 0.0597, wR2 = 0.1468	R1 = 0.0601<br>wR2 = 0.1631	R1 = 0.0545<br>wR2 = 0.1469
R indices (all data)	R1 = 0.0662, wR2 = 0.1600	R1 = 0.0751<br>wR2 = 0.1825	R1 = 0.0576<br>wR2 = 0.1539
Largest diff. peak and hole [e Å^{-3}]	0.380 and −0.620	0.596 and −0.549	0.589 and −0.437
